# Efficacy and Safety of AirFloss Ultra With Essential Oils Versus Waxed Dental Floss as Adjunct to Toothbrushing: A Randomised Controlled Clinical Trial

**DOI:** 10.1111/idh.70010

**Published:** 2025-12-12

**Authors:** Tim M. J. A. Thomassen, Dagmar E. Slot, Therese A. Elkerbout, Eveline van der Sluijs, Fridus A. Van der Weijden

**Affiliations:** ^1^ Department of Periodontology Academic Centre for Dentistry Amsterdam (ACTA) University of Amsterdam and Vrije Universiteit Amsterdam Amsterdam the Netherlands

**Keywords:** AirFloss, dental floss, dental plaque, gingival abrasion, gingival bleeding, gingivitis

## Abstract

**Aim:**

This study aims to determine the efficacy and safety of AirFloss Ultra with essential oils (AFeo) compared to waxed dental floss (DF) as an adjunct to regular manual toothbrushing.

**Material and Methods:**

This study employed a randomly assigned, parallel, examiner‐blind design. Eighty‐two systemically healthy, right‐handed volunteers with ≥ 25% gingival bleeding in the lower jaw but without periodontitis were enrolled. After 3 weeks of experimentally induced gingivitis, gingivitis reversal was assessed over the following 4 weeks. Subjects were divided into two groups: one used AFeo in addition to twice‐daily manual toothbrushing with fluoride toothpaste, while the other used DF with the same brushing regimen. Bleeding On Marginal Probing (BOMP), Modified Silness and Löe Plaque Index (MPI), and approximal gingival abrasion scores (GAS) were recorded. These clinical parameters were analysed at baseline, intermediate time points, and study conclusion using appropriate parametric and non‐parametric statistical methods.

**Results:**

The AFeo and DF groups showed a reduction in mean BOMP, MPI, and GAS scores. There were no significant differences in these parameters between the AFeo and DF groups at any time point (*p* > 0.05). Furthermore, no serious adverse events were reported.

**Conclusion:**

No significant difference in efficacy was observed between the AirFloss Ultra filled with an essential oil solution and waxed dental floss when used alongside manual toothbrushing. This conclusion is based on assessments of gingival bleeding, dental plaque, and gingival abrasion scores in the lower jaw following experimentally induced gingivitis. Both products were also found to be safe for use.

## 1 Introduction

Periodontal diseases manifest across a spectrum of severity, from mild gingival inflammation to profound disability [1, 2]. The two main types of periodontal disease are plaque‐induced gingivitis and periodontitis. The pathological changes associated with these diseases can impair masticatory and phonetic functions, leading to compromised aesthetics and social interactions [1, 2, 3, 4]. Gingivitis comprises an inflammatory lesion arising from the interactions between dental plaque, or biofilm, and the host's immune‐inflammatory response. The condition's reversible nature is contingent upon the reduction of dental plaque levels, both supra‐gingivally and sub‐gingivally. Gingivitis is a major risk factor and foundational precursor to periodontitis [5]. Consequently, the effective management of gingivitis is the primary preventive measure against the development of periodontitis [6]. Several methods are available for mechanically removing dental plaque from teeth, with toothbrush use widely regarded as the most effective [7, 8, 9, 10].

While a toothbrush effectively removes plaque from buccal, lingual, and occlusal surfaces, it does not thoroughly clean the interdental area if exposed to biofilm growth on such surfaces [6]. Several factors influence the selection of an appropriate interdental cleaning aid, including ease of use, interdental space size, acceptability, dexterity, and motivation [11]. While there is no one‐size‐fits‐all solution, a desirable interdental cleaning device should be user‐friendly and efficient in plaque removal and should not harm soft or hard tissues [12].

Dental floss is the most commonly used interdental cleaning device for removing plaque between teeth [13]. However, the product has disadvantages for some patients, including those with open interdental embrasure spaces, those who lack motivation, and those with limited manual dexterity. Because dental floss is technique‐sensitive, it often requires detailed demonstration by clinicians to be effective. Based on the synthesis of systematic reviews, a meta‐review on the efficacy of interdental mechanical plaque control in managing gingivitis concluded that the majority of existing studies did not provide substantial evidence supporting the general effectiveness of flossing in plaque removal [12]. This finding corroborates an earlier systematic review suggesting that dental care professionals should individually assess each patient's ability to floss effectively [14].

Oral irrigation for home use has been extensively studied in laboratory and clinical settings since the early 1960s. Among oral care devices, oral irrigators (OIs) are some of the most widely researched [15, 16, 17]. In general, OIs are designed to remove plaque and soft debris through the mechanical action of a jet stream of water. OIs have diverse designs, features, and functionalities. Some models have multiple pressure settings, specialised tips, and built‐in timers [18, 19, 20]. These devices may use a continuous water stream or incorporate pulsating technology to enhance their cleaning effectiveness. A more novel type of OI, the Philips Sonicare AirFloss Ultra (AF) from Royal Philips N.V., delivers quick bursts of air and microdroplets that reach between teeth and remove interproximal plaque biofilm. De Jager et al. [21] evaluated the adjunctive effect to toothbrushing in adults with moderate gingivitis during a single‐blind, four‐week, parallel, randomised controlled clinical trial. They concluded that the novel interproximal cleaning device was safe to use, removed more interproximal plaque in single‐use, and significantly reduced gingivitis over 4 weeks, than manual toothbrushing alone [21]. The device also increased oral care compliance since 96% of irregular flossers reported using the AF device four or more days per week [22]. Another potential advantage of the AF device is that due to its efficient use of liquid, it can cost‐effectively be used with fluids other than water, such as a mouth rinse containing antimicrobial properties [23].

Mouthrinses have been used for centuries for medicinal and cosmetic purposes. The efficacy of their chemical ingredients has been extensively researched through scientific studies and clinical trials [24]. Essential‐oils mouthwash (EOMW) has the longest history of use, originating in the 19th century. These mouthrinses have been specifically used as a preventive measure against dental and periodontal diseases. A systematic review [25] established EOMW's anti‐gingivitis effect when used as an adjunct to unsupervised oral hygiene rather than a placebo or control. It appears that EOMW provides a significant oral health benefit over 6 months of use [26, 27].

The AF has primarily been tested using water in its reservoir. Combining the microburst technology with an active chemical agent deserves further evaluation. Therefore, this study compares the efficacy of an EOMW used in the AF device (AFeo) compared to waxed dental floss (DF) as an adjunct to regular manual toothbrushing and their impact on gingival bleeding, dental plaque, and gingival abrasion. Additionally, soft and hard tissue safety assessments were evaluated.

## 2 Materials and Methods

The recommendations for strengthening reporting as presented in the Consolidated Standards of Reporting Trials (CONSORT) [28] and the Template for Intervention Description and Replication (TIDieR) [29] were followed. This study observed the Good Clinical Practice (CPMP/ICH/135/95) guidelines, in agreement with the ethical principles of the Declaration of Helsinki (seventh revision, 2013, Brasil) and in accordance with the Medical Research Involving Human Subjects Act (WMO) and applicable local regulations. The study was approved by the medical ethical committee at Amsterdam Medical Centre (2014_374#8201545) and is registered with the WHO (World Health Organization) (International Clinical Trials Registry Platforms under registration number [NL‐OMON29183]) (online Appendix A).

The study was conducted in 2015 at the Department of Periodontology Academic Centre for Dentistry of Amsterdam (ACTA), the Netherlands. Before enrolment, the volunteers were given verbal and written information regarding the study's aim, rationale, and duration. Details of the trial and the potential risks involved were outlined. Subsequently, eligible subjects signed an informed consent form with the understanding that they could withdraw from participation at any point. The recruitment, screening, familiarisation, experimental, and treatment phases took 2.5 months.

### 2.1 Objectives

#### 2.1.1 Primary Objective

The primary objective was to assess the efficacy of AirFloss Ultra with essential oil mouthwash, compared to waxed dental floss, as adjunctive therapies to manual toothbrushing in a healing of experimental gingivitis model [30] over a four‐week treatment period, as assessed by the Bleeding On Marginal Probing (BOMP) index [31, 32, 33] on the lower jaw.

#### 2.1.2 Secondary Objectives

- To compare AFeo with DF in the reduction of surface plaque over a four‐week treatment period, as assessed by the Van der Weijden [34, 35] modification of the Silness and Löe Plaque Index [36].
- To compare the number of gingival abrasions between AFeo and DF during a four‐week treatment period following experimental gingivitis, as assessed by the Gingival Abrasion Score [37, 38, 39, 40].
- To describe the study participants' perceptions of the two interdental devices.

### 2.2 Name and Description of (Non‐) Investigational Products

#### 2.2.1 The Investigational Products

- AFeo: (Philips Sonicare AirFloss Ultra + Listerine Cool Mint mouth rinse, Johnson & Johnson, Skillman, NJ).
  ○ Active Ingredients: Eucalyptol 0.092%, Menthol 0.042%, Methyl Salicylate 0.060%, Thymol 0.064%.
  ○ Inactive Ingredients: Water, Alcohol (21.6%), Sorbitol, Poloxamer 407, Benzoic Acid, Sodium Saccharin, Sodium Benzoate, Flavour, Green 3.
- DF: Waxed dental floss, Johnson & Johnson, Type: Ultraclean.
  ○ If necessary, participants were instructed to use a floss thread needle, which can be applied to pass the floss thread below the contact point (e.g., with a lingual retention wire in place). Floss Threaders, Brand: SUNSTAR GUM, Type: Eez‐Thru.

#### 2.2.2 The Non‐Investigational Products

- Manual toothbrush Lactona iQ Soft, Lactona Europe B.V. Bergen op Zoom, the Netherlands.
- Regular fluoride toothpaste HEMA toothpaste, HEMA Nederland B.V., the Netherlands.
  ○ Content: tubes de 75 mL containing 1450 ppm fluoride.
  ○ Ingredients: aqua, sorbitol, hydrated silica, sodium lauryl sulfate, titanium dioxide, xanthan gum, aroma, cetylpyridinium chloride, sodium fluoride, sodium saccharin, sodium benzoate.

### 2.3 Study Design

**FIGURE 1:**
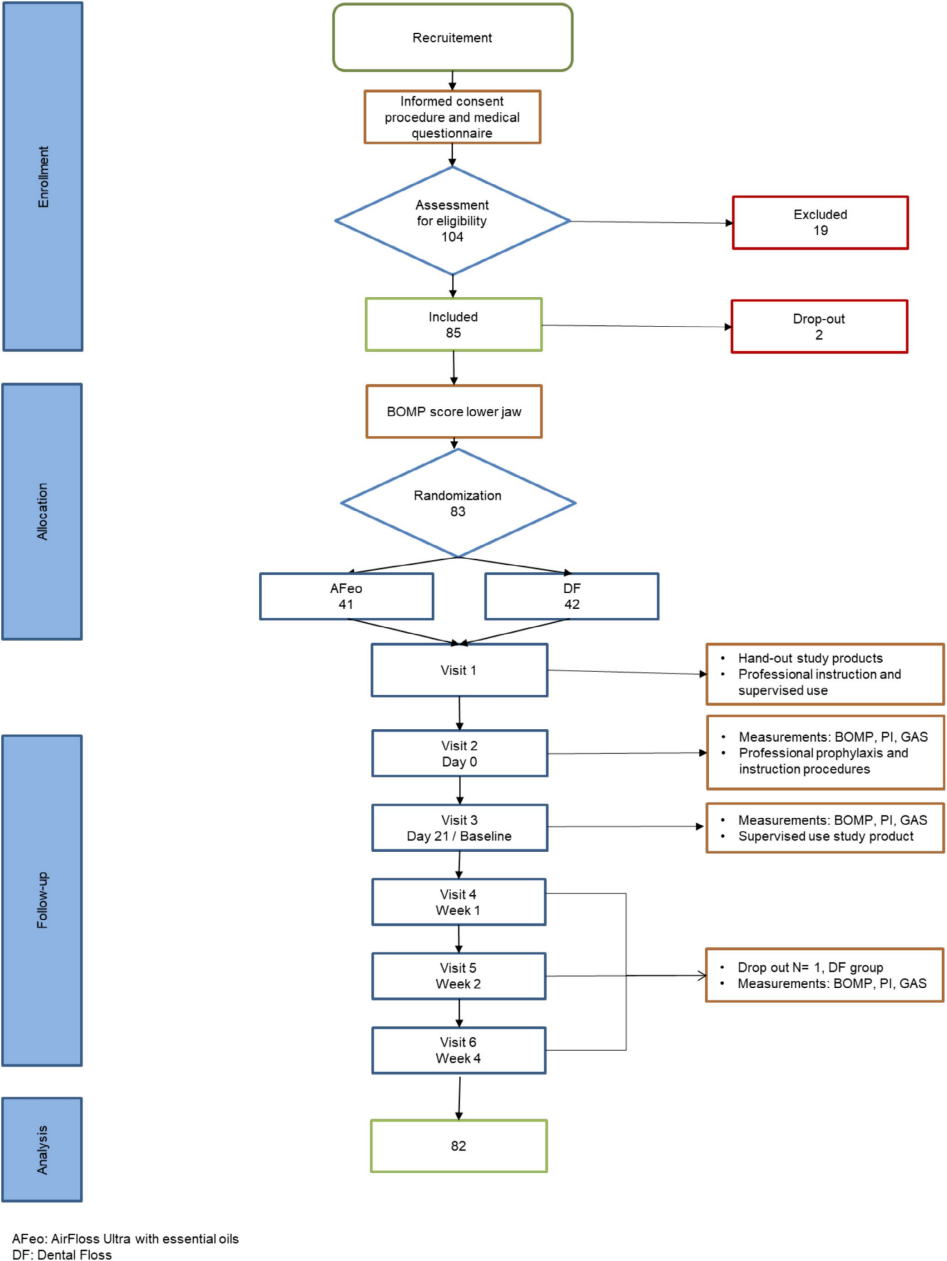
Flow chart.

This study used a parallel, single‐blind (examiner), randomly assigned intervention design evaluating the healing of experimentally induced gingivitis. This model was introduced by Van der Weijden et al. [41] and has been used by other authors to evaluate oral hygiene products [42, 43, 44, 45]. Figure 1 provides a detailed flowchart, while the table of events summarises the procedures (online Appendix Table 1).

### 2.4 Sample Size

This study was designed to detect a difference of 0.16 in the BOMP score between the two randomised groups. Assuming a common standard deviation of 0.25, a sample size of 39 subjects in each of the two treatment arms provides 80% power if the type I error rate is 5% [45].

### 2.5 Randomization, Blinding, and Treatment Allocation

Randomization was performed using true random numbers generated by sampling and processing a source of entropy outside the computer. The source was atmospheric noise, sampled and fed into a computer, avoiding any buffering mechanisms in the operating system (www.random.org). No stratification was applied. Every subject received a unique trial number. Every enrolled subject received a unique trial number. To properly implement allocation concealment the group allocation (AFeo or DF) was placed inside an opaque, tamper‐proof sequentially numbered envelope (SNOSE) and opened only after participant enrollment. The randomization code was kept by the primary investigator in a sealed envelope in the investigator site file, stored in a secured area, and not accessible to the examiners A separate CRF randomization form was used to allocate AFeo or DF. Subjects were instructed not to reveal their group assignment to the clinical examiner.

### 2.6 Study Procedures

#### 2.6.1 Recruitment Phase

Subjects were recruited from a database containing individuals from universities in and around Amsterdam who had subscribed as potentially interested in participating in clinical research. Potential participants were informed about the research by email. Flyers, posters, and advertisements were used to attract additional participants.

#### 2.6.2 Screening Phase

Before screening, a written explanation of the study's background was sent by email to inform the subjects of their involvement and the research objectives. Additionally, participants were requested to give written informed consent before being screened for suitability. The subjects completed a medical questionnaire in order to be classified as systemically healthy. Two dental hygienists (NHH and EVDS) performed an oral examination to screen potential subjects to ascertain whether they had a ≥ 25% BOMP in the lower jaw and Dutch Periodontal Screening Index [46, 47] (DPSI) between 0 and 3 minus (online Appendix B). According to the 2017 EFP/AAP classification, these subjects could be classified as having ‘gingivitis’ [2]. Dental floss had to fit interdentally in at least three interdental spaces per quadrant in the lower jaw, excluding the interdental central incisor space. Of these three spaces, at least two had to involve molar areas. Participants were excluded if they had overt dental caries, orthodontic appliances, removable (partial) dentures, oral and/or peri‐oral piercings, apparent oral lesions, or were pregnant. Additionally, participants were excluded if they had participated in a clinical trial in the previous 30 days. For a detailed list of inclusion and exclusion criteria, see online Appendix Table 2.

#### 2.6.3 Familiarisation Phase—Visit 1

Gingival bleeding (BOMP) [31, 32, 33] scores in the lower jaw were assessed (NHH). A dental hygienist (DES) performed professional instruction followed by supervised use of the study products. Subjects were instructed to use the interdental device according to the randomization assignment. The instructions were delivered in an area separated from the examiners to ensure blinding. A treatment package was provided, containing:

- A standard manual toothbrush
- A standard tube of toothpaste
- AFeo or DF, according to randomization
- Appropriate written instructions for product A or B

Subjects were required to complete a calendar to record their daily use of the assigned product to check compliance. They were instructed to do this for every upcoming phase and bring the calendar to each visit.

#### 2.6.4 Experimental Gingivitis Phase—Visit 2 (Day 0)

Interdental devices and calendars of use for the familiarisation phase were collected by the study coordinator (TAE). Gingival bleeding (NHH), dental plaque scores (NHH), and gingival abrasion (EVDS) scores were calculated for the lower jaw. The same trained and calibrated examiners performed the examinations throughout the study. A dental hygienist then gave the participants a professional prophylaxis in the lower jaw, creating a dentition free of plaque, stain, and calculus, as described by Slot et al. [48]. The subjects were instructed to refrain from brushing the lower jaw for the next 21 days and to use a toothbrush and toothpaste only for the upper jaw. After 21 days, the subjects returned to the clinic for the start of the treatment phase.

#### 2.6.5 Treatment Phase—Visit 3–6 (Week 1 to 4)

Gingival bleeding, dental plaque scores, and gingival abrasion scores were assessed for the lower jaw. Subjects were instructed to use the interdental device according to their randomization assignment (Product A or Product B), with instructions and supervised use conducted in a separate area to ensure blinding. During the treatment phase, subjects returned for assessments at Visits 3, 4, and 5, where gingival bleeding (NHH), dental plaque scores (NHH), and gingival abrasion (EVDS) scores were recorded for the lower jaw. The participants then returned for Visit 6, where these assessments were repeated, alongside querying subjects' attitudes toward the study products via questionnaire. A detailed description of the flowchart containing patient information can be found in online Appendix Figure 1.

### 2.7 Outcomes Variables

#### 2.7.1 Gingival Inflammation

Lower jaw gingival inflammation was measured using the BOMP score [31, 32, 33]. In this method, a bleeding score (0 = no bleeding, 1 = pin prick, and 2 = excess) was given to six areas of each tooth after probing, for which a WHO‐approved ball‐ended probe was used (Ash Probe EN15, Dentsply International, York, PA, USA). An average score was calculated for each subject by summing the scores from all the sites and dividing this figure by the number of scorable sites.

#### 2.7.2 Surface Plaque

Dental plaque was assessed using the Van der Weijden et al. modification [34] of the Silness and Löe Plaque Index [36]. Six surfaces on each tooth on the lower jaw were scored on a scale ranging from 0 to 3, depending on the thickness of the plaque. A summary index score was calculated for each subject by summing the scores from all the sites and dividing this figure by the number of scorable sites.

#### 2.7.3 Gingival Abrasion

The gingival abrasion score on the lower jaw was assessed using the Gingival Abrasion Score (GAS) [37, 38, 39, 40]. For each subject, the gingiva's three areas (interdental, cervical, or midgingival) were assessed for abrasions. Observed lesions were grouped by size based on their largest width, as follows:

- Small: If largest width of lesion ≤ 2.5 mm,
- Medium: If 2.5 mm < largest width of lesion ≤ 5 mm
- Large: If largest width of lesion > 5 mm

Sites were defined as non‐evaluable if teeth were missing or approximal restorations were present.

For each follow‐up visit, the reduction and percent reduction in BOMP [31, 32, 33], plaque [34, 35], and GAS [37, 38, 39, 40] were calculated as follows: The follow‐up score was subtracted from the baseline score for each site; this difference was averaged and divided by the total number of scorable sites, and percent reduction was calculated by dividing the reduction by the baseline score and multiplying this by 100%.

A detailed description of the outcome variables and their scoring method can be found in online Appendices B–E. In addition, the patient instruction forms are provided in online Appendices F and G. No specific instructions on toothbrushing were provided. Participants were allowed to continue their habitual methods.

### 2.8 Safety Analysis

Safety evaluations were performed by recording clinical adverse events at the time originally reported and at each visit thereafter.

### 2.9 Statistical Analysis

This study's primary analyses focused on BOMP [31, 32, 33] measurements, while secondary parameters encompassed dental plaque [34, 35] and gingival abrasion [37, 38, 39, 40], all in the lower jaw. The total mean and standard deviation for these clinical parameters were analysed across all subjects at baseline, intermediate time points, and at the end of the study. Additionally, 95% confidence intervals were computed. Furthermore, the incremental changes from baseline values were analysed for these parameters. Parametric and non‐parametric statistical methods were employed as appropriate for a comprehensive understanding of the dataset. Statistical significance was determined with a threshold of *p* < 0.05. Boxplots were utilised to visually compare the distribution of BOMP, MPI, and GAS scores across groups at different time points, displaying the median, interquartile range (IQR), and potential outliers. All analyses were conducted using SAS software.

## 3 Results

### 3.1 Participants

Of the 104 participants screened, 19 were excluded based on the eligibility criteria. Two participants were enrolled but not randomised, and one did not finish the full study protocol (Figure 1). In total, 82 participants—47 females (57%) and 35 males (43%) with a mean age of 22.9 (SD 3.3) years—completed the study and were included in the analyses (Table 1). There were no differences at baseline between the groups for the primary outcome, BOMP [31, 32, 33], and the secondary outcomes, MPI and GAS (Table 1).

**TABLE 1:** Baseline demographics and variables.

Parameter		Mean (SD)			
		Total (*N* = 82)	AFeo (*N* = 41)	DF (*N* = 41)	*p‐value*
Age (years)^a^		22.9 (3.3)	22.6 (2.7)	23.2 (3.8)	0.391
Sex^b^	Female (%)	47 (57.3%)	24 (58.5%)	23 (56.1%)	0.823
	Male (%)	35 (42.7%)	17 (41.5%)	18 (43.9%)	
# of missing teeth^a^		0.3 (0.9)	0.4 (1.1)	0.2 (0.8)	0.485
BOMP^a^		1.58 (0.27)	1.55 (0.31)	1.62 (0.24)	0.271
MPI^a^		1.99 (0.08)	1.99 (0.09)	1.98 (0.07)	0.939
GAS^a^		5.95 (4.01)	6.44 (3.78)	5.46 (4.21)	0.273

*Note: p* < 0.05 indicates statistically significant differences between groups.

Abbreviations: AFeo, AirFloss with essential oils; BOMP, Bleeding On Marginal Probing Score; DF, waxed dental floss; GAS, gingival abrasion score; MPI, marginal probing index.

^a^ One‐way analysis of variance (ANOVA).

^b^ Chi‐square test.

### 3.2 Primary Outcome

Table 2 displays the values of BOMP scores [31, 32, 33] at baseline (Day 0) and during the treatment phase (Weeks 1, 2, and 4) for the AFeo and the DF group. The mean reduction in BOMP scores [31, 32, 33] from baseline for the AFeo group was 0.60 (SD 0.32) at Week 4, while for the DF group, the mean reduction was 0.70 (SD 0.32). The analysis revealed a statistically significant difference between baseline and Week 4 for both groups. There were no statistically significant differences between the groups at any time point (Table 2).

**TABLE 2:** Bleeding On Marginal Probing (BOMP) scores for both treatment modalities.

BOMP	Mean (SD)				Difference Day 0‐week 4	*p‐value* ^a^
	Baseline	Treatment phase				
*N* = 82	Day 0	Week 1	Week 2	Week 4		
AFeo	1.55 (0.26)	1.14 (0.26)	0.99 (0.32)	0.95 (0.32)	0.60 (0.32)	**< 0.01**
DF	1.62 (0.26)	1.08 (0.26)	1.00 (0.32)	0.92 (0.32)	0.70 (0.32)	**< 0.01**
Difference	−0.07 (0.38)	0.06 (0.38)	−0.01 (0.45)	0.03 (0.45)	−0.10 (0.07)	0.17
*p‐value* ^a^	0.27	0.37	0.87	0.72	0.17	

*Note: p* < 0.05 indicates statistically significant differences between groups.

Abbreviations: AFeo, AirFloss with essential oils; DF, waxed dental floss.

^a^ One‐way analysis of variance (ANOVA).

The boxplot (online Appendix Figure 2) of the BOMP score [31, 32, 33] showed that the median scores increased slightly in both groups from the familiarisation to baseline periods, followed by stabilisation over subsequent weeks. The groups' IQR boxes overlapped across all visits. Both groups showed several outliers at each time point, with no difference in the pattern or number of outliers. The whiskers extended to the smallest and largest values within 1.5 times the IQR from the first and third quartiles. No visible difference in the length of the displayed whiskers was present.

### 3.3 Secondary Outcomes

Table 3 shows the mean (SD) values of the MPI [34, 35] and GAS [37, 38, 39, 40] for both treatment modalities. The statistical analysis results indicate a statistically significant difference between baseline and Week 4 scores for both treatment modalities. Furthermore, there were no statistically significant differences in MPI [34, 35] and GAS scores [37, 38, 39, 40] between the AFeo and DF groups at any time point. Online Appendix Figures 3 and 4 display boxplots illustrating these indices for both groups, confirming the observations for the primary parameter. The same pattern was observed when examining reductions relative to baseline scores. For both indices, no statistically significant differences were observed between the AFeo and DF groups at any time point (Table 3). The mean appreciation score for AFeo was 6.06 (SD = 2.09), while for DF, it was 5.62 (SD = 2.21). An independent samples *t*‐test showed no statistically significant difference between the two groups (*t*(78) = 0.91, *p* = 0.365).

**TABLE 3:** Modified Silness and Löe Plaque Index (MPI) and gingival abrasion score (GAS) for both treatment modalities.

*N* = 82	Mean (SD)				Difference Day 0‐week 4	*p‐value* ^a^
	Baseline	Treatment phase				
	Day 0	Week 1	Week 2	Week 4		
MPI						
AFeo	1.99 (0.06)	1.08 (0.51)	1.03 (0.51)	0.95 (0.45)	1.04 (0.07)	**< 0.01**
DF	1.98 (0.06)	1.22 (0.51)	1.18 (0.51)	1.08 (0.45)	0.90 (0.07)	**< 0.01**
Difference	0.00 (0.09)	−0.14 (0.72)	−0.15 (0.72)	−0.13 (0.63)	0.14 (0.07)	0.19
*p‐value* ^a^	0.94	0.20	0.19	0.26	0.19	
GAS						
AFeo	6.44 (3.97)	13.49 (5.63)	15.59 (6.78)	19.33 (7.42)	12.89 (0.92)	**< 0.01**
DF	5.46 (3.97)	13.00 (5.63)	15.32 (6.78)	19.75 (7.42)	14.29 (0.92)	**< 0.01**
Difference	0.98 (7.99)	0.48 (11.33)	0.27 (13.70)	−0.42 (14.87)	1.40 (2.04)	0.49
*p‐value* ^a^	0.273	0.700	0.858	0.800	0.49	

*Note: p* < 0.05 indicates statistically significant differences between groups.

Abbreviations: AFeo, AirFloss with essential oils; DF, waxed dental floss.

^a^ One‐way analysis of variance (ANOVA).

### 3.4 Adverse Events

Six study‐related adverse events were reported following dental floss use, encompassing gum irritation, soreness, gingival lesions, pain, and redness and swelling in the lower buccal gingiva. No serious adverse events were reported.

## 4 Discussion

### 4.1 Summary of Key Findings

This study compared the efficacy of AFeo to DF as an adjunct to regular MTB in relation to gingival bleeding, dental plaque, and gingival abrasion. The primary outcome, measured by the BOMP scores, showed no statistically significant differences between the AFeo and DF groups at any point. Both groups exhibited a gradual reduction in BOMP scores from baseline (after 3 weeks of experimental gingivitis) over Weeks 1 to 4. Additionally, secondary outcomes assessed using the MPI and GAS showed no significant differences between the AFeo and DF groups at any point. Mwatha et al. [23] compared the efficacy of three interproximal cleaning methods (DF, AFeo, and AF used with BreathRx) as adjuncts to MTB versus MTB alone in relation to gingivitis. It was concluded that among these interproximal cleaning methods, AFeo and AF used with BreathRx provided a comparable reduction in gingivitis and plaque to DF and were found to be safe for oral tissues [23]. These findings corroborate the results of the present study. The impact of incorporating an essential oil mouthwash into its reservoir remains uncertain. Therefore, a comparative study of AF with and without essential oils is warranted (see Section 4.6).

### 4.2 Study Design

Experimental gingivitis, as originally described by Löe et al. [49], is a controlled clinical model used to study the onset and progression of gingival inflammation in response to dental plaque accumulation. The principle of experimental gingivitis is that, in periodontally healthy participants, gingivitis develops between 10 and 21 days of refraining from mechanical plaque control when starting from a state of gingival health and no clinically detectable plaque. This model has been used extensively to understand the pathophysiology of gingivitis and evaluate the efficacy of various oral hygiene products and interventions [30, 43, 44, 45].

A modification of this study model, where participants refrained from oral hygiene for 21 days to allow for the development of gingivitis before commencement of treatment [41], has not been used in evaluating the efficacy of AFeo before. Van der Weijden et al. [41, 50] introduced this multiphase model to assess short‐term gingivitis studies, aiming to overcome challenges associated with earlier long‐term gingivitis studies [32, 33]. Initially, there was a two‐week familiarisation period during which participants practiced with toothbrushes and received professional instructions. This phase led to a clear reduction in bleeding, attributed to the professional instruction and training, which impacted the level of gingivitis. To re‐establish a baseline level of inflammation, an experimental gingivitis phase was introduced as the second phase of the study. The primary experiment then began, focusing on the healing of experimentally induced gingivitis. Participants resumed toothbrushing, leading to a gradual reduction in gingivitis over time, as anticipated. The healing of experimentally induced gingivitis is a valuable short‐term study design [51].

Experimental gingivitis models are typically short‐term studies that focus on early‐stage inflammation. As a result, they may not fully capture the long‐term effects of plaque accumulation and chronic gingival inflammation. Additionally, individual differences in genetic, immunological, and microbiological factors can lead to variability in how subjects respond to plaque accumulation [52].

Ensuring participant compliance with study protocols, such as refraining from oral hygiene practices, can be challenging and may influence the study results. To address these challenges, a calendar was used to remind and motivate participants to adhere to the study regimen, minimising variability and improving the results' reliability. To minimise patient discomfort and address aesthetic concerns, experimental gingivitis was induced exclusively in the lower jaw. This approach was based on Putt et al. [53], who demonstrated that a partial‐mouth experimental gingivitis model permits the development of plaque and gingivitis comparable to whole‐mouth studies where oral hygiene was suspended for 3 weeks.

For assessing plaque scores, the Modified Silness and Löe Plaque Index [34, 35] was used. The Silness & Löe Plaque Index [36] was modified as described by Van der Weijden et al. [34] to assess six surfaces per tooth instead of the original four. The evaluated surfaces include distal‐buccal, buccal, mesial‐buccal, distal‐lingual, lingual, and mesial‐lingual. Each surface is assigned a score of 0, 1, 2, or 3 based on defined criteria. This modified index is particularly well‐suited for studies focusing on the interdental area, as interdental surfaces contribute up to two‐thirds of the total plaque score. In contrast, plaque area indices such as the Quigley and Hein Index [54] are less appropriate for this purpose, as they are weighted more heavily toward vestibular and lingual surfaces rather than interproximal areas.

### 4.3 AirFloss versus Dental Floss

Among various methods for removing interproximal plaque, dental floss is the most frequently recommended. Berchier et al. [14] assessed the effectiveness of DF combined with toothbrushing on plaque and gingivitis in adults. Their systematic review (SR) and meta‐analysis found that DF provided no benefit beyond toothbrushing in removing plaque and reducing gingivitis. A Cochrane SR by Sambunjak et al. [55] included various floss‐related products and concluded there was weak evidence suggesting that DF in addition to toothbrushing reduced gingivitis compared to toothbrushing alone. Another SR [56] reported that DF effectively reduced the risk of interproximal caries only when performed professionally. Self‐performed flossing did not show a beneficial effect [56].

Given this evidence regarding dental floss (DF), an alternative interpretation of the present findings could be that if DF is ineffective and there is no statistically significant difference between the AFeo and DF results, the AFeo may not provide a beneficial effect. However, previous research has shown that combining AF with MTB significantly improves interproximal plaque scores and leads to greater reductions in gingivitis scores after 2 and 4 weeks compared to toothbrushing alone [21, 23]. Additionally, it is incorrect to assert that DF provides no benefit. A study by Barendregt et al. [57], which included a 21‐day experimental gingivitis protocol, compared no treatment to using DF alone. The results demonstrated that in a well‐instructed population, the treatment group exhibited an almost 50% reduction in the development of gingivitis. However, due to a lack of scientific evidence supporting routine flossing, it is left to the dental care professional to determine on an individual patient basis whether high‐quality flossing is an achievable goal [14].

### 4.4 AirFloss Working Mechanism

Fluid shear stress is an alternative mechanical strategy for biofilm control [58]. Previous research indicates that sufficiently high fluid shear stress can induce biofilm detachment [59, 60, 61, 62]. Both high‐velocity water droplets [63] and entrained air bubbles [64, 65] have demonstrated efficacy in removing bacteria and biofilms from surfaces, utilising the surface‐tension force generated by the passage of an air/water interface [66]. A notable advantage of utilising fluid forces for biofilm removal is their ability to extend mechanical action beyond the device itself, leveraging currents generated in the fluid surrounding teeth through water jets produced by oral irrigation [67].

Oral irrigators (OIs) have been extensively studied in laboratory and clinical research. A recent network meta‐analysis by Kotsakis et al. [68] positioned power‐driven water flossers as the second most effective adjunct to toothbrushing, following interdental brushes. Oral irrigators can also be employed to apply antimicrobial agents for chemical plaque control [17, 69, 70, 71]. Adjunctive use of OI with antimicrobial agents can effectively reduce clinical and microbiological parameters in individuals with gingivitis [69]. Supragingival irrigation with chlorhexidine, in particular, demonstrated significant efficacy in this regard [17, 69, 71, 72, 73].

Various theories propose mechanisms by which OIs may lower bleeding indices without significantly impacting plaque levels, including alterations in biofilm composition, interference with plaque maturation, immune response stimulation, and reduction in plaque thickness [68, 70, 74]. The disadvantage of using an OI is that continuous water jets require large reservoirs and can be messy to use due to the large volumes of water involved. Therefore, the AF incorporates specific air and micro‐droplet technology. The mechanism of the AF differs from other OIs in several aspects. While OIs use a jet stream of water at low velocity, the AF shoots a microdrop volume of water and entrained air at high velocity into the interdental space in discrete bursts [75, 76]. This method creates high wall shear stress and high‐impact pressure over short periods, minimising water volume and reducing cleaning times.

### 4.5 Value‐Based Oral Health Care and Patient Preferences

Prevention of periodontitis and caries at the individual level necessitates diligent oral hygiene, minimising dietary sugar intake and eliminating tobacco use [77]. The World Health Organization [78] advocates for straightforward, safe, and cost‐effective strategies to enhance oral health following these principles. For personal dental care, WHO advises brushing teeth twice daily with fluoride toothpaste containing 1000–1500 ppm fluoride [79]. Additionally, interdental cleaning should be performed at least once daily using appropriate tools such as dental floss, interdental brushes, or irrigators [79]. Individuals and dental care professionals need to implement cost‐effective measures focused on preventive care [78].

In this regard, the concept of value‐based healthcare (VBH), introduced by Porter [80], emphasises cost‐effectiveness. This concept has become integrated into the medical field, particularly in Western societies. Value‐based ‘oral’ health care (VBOHC) [81] focuses on improving oral health outcomes relative to costs, encapsulated by the phrase “patient health outcomes achieved per dollar spent.” To date, no value‐based analysis of interdental cleaning has been conducted. Several factors need to be considered for such an analysis. First, the cost of interdental cleaning aids, such as the AF, is significantly higher than that of DF, particularly when essential oils are included in the device's reservoir. Additionally, the AF is available in various models with different price points. Second, it is important to determine the expected improvement in plaque removal and the resulting preventive effects on gingival health and the risk for caries. By conducting a thorough analysis based on these considerations, VBOHC can provide insights into the specific oral health outcomes achievable for an individual using an interdental cleaning aid. This information will help determine whether the financial investment in an AF with essential oils is justified and beneficial for everyone. This kind of analysis is valuable for future research, as it can guide more effective resource allocation and personalised oral health interventions. A cautious prediction from a financial perspective suggests that the AFeo offers limited additional value compared to DF, as this study indicates no statistically significant difference in the device's effectiveness.

Although finances are an important consideration when choosing the most suitable interdental cleaning device, patient satisfaction and preferences are also significant factors. In this study, there was no difference in the appreciation of the AFeo and DF by the participants. In a recent prospective cohort study involving periodontitis patients undergoing regular supportive periodontal therapy, patient satisfaction with the AF was high, with approximately 80% expressing overall satisfaction [82]. None of the study's participants reported discomfort or pain during device use, indicating that the water burst pressure was well tolerated. Additionally, over 75% of the participants indicated a willingness to carry the device while travelling and to recommend it to family members and friends. This finding aligns with Heiß‐Kisielewsky et al. [83], who evaluated dental students' preferences and found that 82% found the device easy to use and 59% preferred it over flossing. Another study explored patients' preferences for interdental cleaning methods, focusing on those who use floss irregularly [22]. In this sample, the AF was favoured over other modalities, receiving higher ratings for ease of use than traditional floss or oral irrigators. The AF was also noted as being gentler on teeth and gums and offering better access to the posterior areas of the mouth than string floss.

These findings suggest that such devices could be beneficial for patients with lower motivation or compliance and those with reduced dexterity or difficulty managing other types of interdental cleaning devices [82].

### 4.6 Bacterial Colonisation

Using oral irrigation devices like the AF poses a risk of bacterial colonisation and biofilm formation. Due to contact with the oral environment, the nozzle tip is susceptible to bacterial colonisation, potentially leading to biofilm formation within the nozzle and the device. Biofilm buildup is common in water pipework systems, where water circulates from the device's reservoir through internal channels to the nozzle. A proof‐of‐principle study by Bertl et al. [84] demonstrated bacterial colonisation of the AF nozzle and device after 3 weeks of daily use, with aerobic and anaerobic species transmitted through the water jet. Another study [85] by the same research group found that using the device with antimicrobial mouthrinse or cleaning it did not promote disinfection or effectively prevent the growth of bacteria, particularly *S. mutans* , a caries‐associated pathogen. The exchange of nozzles did not mitigate the risk of cross‐contamination. Therefore, the authors recommend one device per person to minimise the risk of cross‐contamination within households [85].

### 4.7 Limitations and Recommendations

- One limitation of this study is the inability to blind participants to the device used, which may have influenced their behaviour toward the novel device. This introduces the risk of the Hawthorne effect, wherein individuals modify their behaviour simply because they are aware of being observed In this context, participants who used the AirFloss device may have been more motivated to perform optimally compared to those using dental floss. This potential bias should be considered by the reader when interpreting the results.
- Future studies may consider incorporating an assessment of manual dexterity to better understand its potential impact on study outcomes. However, given the randomised controlled trial design, it is a reasonable assumption that differences in manual dexterity were equally distributed across study groups.
- The use of a floss thread needle when necessary is a limitation, as it does not reflect typical flossing behaviour. This may affect the generalizability of the results and should be considered when interpreting the findings.
- A limitation of this study is that we evaluated both the mechanical and antiseptic action of the Airfloss Ultra with essential oils and water (EOMW), while only the mechanical action of waxed floss was assessed. This difference in evaluation should be considered when interpreting the results.
- Although the product under study may no longer be available and has been replaced by newer models, the unchanged mode of action ensures that the study results remain relevant and significant for future product development. Newer models may incorporate advancements in design, power output, or nozzle technology that could influence factors such as cleaning efficiency, pressure distribution, and user experience. Variations in power settings or fluid dynamics might lead to differences in plaque removal efficacy or overall ease of use. Therefore, future research could explore how these refinements affect clinical outcomes in comparison to earlier models.

## 5 Conclusion

There is no significant difference in the efficacy of the adjunctive effect to toothbrushing of the Airfloss Ultra filled with an essential oil solution compared to waxed dental floss. This applies to the gingival bleeding, dental plaque, and gingival abrasions assessed in this reversal of the experimental gingivitis model. Both products were found to be safe for use.

## 6 Clinical Relevance

### 6.1 Scientific Rationale for the Study

Effective interdental cleaning helps prevent gingivitis and dental caries. Dental floss (DF) is often recommended but has limitations in user compliance and effectiveness. This study compares the efficacy of AirFloss Ultra with essential oils (AFeo) and dental floss (DF) in improving gingival health.

### 6.2 Principal Findings

As adjuncts to manual toothbrushing, AFeo and DF improve gingival health after 3 weeks with no significant differences between the groups for BOMP, plaque, or gingival abrasion scores.

### 6.3 Practical Implications

The AFeo could be a viable alternative to traditional floss, particularly for individuals with limited manual dexterity or lack of motivation for regular flossing.

## Author Contributions

All authors have approved the final version of this manuscript before submission and agreed to be accountable for all aspects of the work, ensuring that questions related to the accuracy or integrity of any part of the work were appropriately addressed and resolved. T.M.J.A.T.: contributed to the design, analysis and interpretation, and drafted the manuscript. G.A.W.: contributed as principal investigator to conception and design, analysis and interpretation, and critically revised the manuscript. T.A.E.: participated as clinical research coordinator, and critically revised the manuscript. E.S.: participated as examiner performing gingival abrasion scores, and critically revised the manuscript. D.E.S.: contributed to the conception and design, analysis and interpretation, and critically revised the manuscript.

## Funding

ACTA Research B.V. received financial support from Royal Philips N.V. (Amsterdam, the Netherlands).

## Conflicts of Interest

The study was independently designed. The investigators declare that they have no conflicts of interest. Van der Weijden is the owner of Jardin B.V., which is the former owner of www.ragershop.com (a webshop for various brands of interdental brushes). The study was performed by the Department of Periodontology of ACTA with a commission from ACTA Dental Research B.V. In turn ACTA Research B.V. received financial support from Royal Philips N.V. (Amsterdam, the Netherlands). The Philips Company also provided the study products and assistance with the statistical analysis. The research team of Van der Weijden and Slot at ACTA has previously received either external advisor fees, lecturer fees or research grants from oral health care manufacturers. Those manufacturers included Colgate, Dentaid, Lactona, Procter & Gamble, Sunstar, TePe, and Water Pik.

## Supporting information


**Appendix Table 1:** Table of events.
**Appendix Table 2:** In‐ and exclusion criteria.
**Appendix Figure 1:** Flowchart patient information.
**Appendix Figure 2:** Boxplot presenting Bleeding On Marginal Probing (BOMP) scores from familiarisation to treatment phase.
**Appendix Figure 3:** Boxplot of the Modified Silness and Löe Plaque Index (MPI).
**Appendix Figure 4:** Boxplot of the Gingival abrasion score (GAS).
**Appendix A:** Approval by the medical ethical committee at Amsterdam Medical Centre.
**Appendix B:** Dutch Periodontal Screening Index (DPSI).
**Appendix C:** Gingival inflammation—Bleeding On Marginal Probing (BOMP).
**Appendix D:** Dental plaque—Modified Silness and Löe Plaque Index.
**Appendix E:** Gingival abrasion score.
**Appendix F:** Patient dental floss instruction.
**Appendix G:** Patient AirFloss instruction.

## Data Availability

The data that support the findings of this study are available from the corresponding author upon reasonable request.
