# Supplementary material for: Efficacy and Safety of AirFloss Ultra With Essential Oils Versus Waxed Dental Floss as Adjunct to Toothbrushing: A Randomised Controlled Clinical Trial
Source: Int J Dent Hyg. 2025 Dec 12;24(1):3–15. doi: 10.1111/idh.70010 (PMC12748044; doi:10.1111/idh.70010)

Efficacy and safety of AirFloss Ultra with essential oils vs. waxed dental floss as adjunct to toothbrushing:

- a randomized controlled clinical trial -

Tim M.J.A. Thomassen

Dagmar E. Slot

Therese A. Elkerbout

Eveline van der Sluijs

Fridus A. Van der Weijden

Online Appendices

Online Appendices

Appendix Table 1.
Table of events

Appendix Table 2.
In- and exclusion criteria

Appendix Figure 1.
Flowchart Patient information

Appendix Figure 2.
Boxplot presenting Bleeding On Marginal Probing (BOMP) scores from familiarization to treatment phase

Appendix Figure 3.
Boxplot of the Modified Silness & Löe Plaque Index (MPI)

Appendix Figure 4.
Boxplot of the Gingival abrasion score (GAS).

Appendix A.
Approval by the medical ethical committee at Amsterdam Medical Centre

Appendix B.
Dutch Periodontal Screening Index (DPSI)

Appendix C.
Gingival inflammation – Bleeding On Marginal Probing (BOMP)

Appendix D.
Dental plaque - Modified Silness & Löe Plaque Index

Appendix E.
Gingival abrasion score

Appendix F.
Patient Dental Floss Instruction.

Appendix G.
Patient AirFloss Instruction.

Appendix Table 1. Table of study procedures

Procedure	Performed By:	Screening	Familiarization Phase	Experimental Gingivitis Phase		Treatment Phase		
			Visit 1	Visit 2	Visit 3	Visit 4	Visit 5	Visit 6
			2 Weeks	21 Days		4 Weeks		
				Day 0	Baseline	Week 1	Week 2	Week 4
**Informed Consent**	Investigator	X						
**Medical Dental History & Demographics**	Examiner	X						
**Oral Examination**	Examiner	X	X	X	X	X	X	X
**Dutch Periodontal Screening[1] Index (DPSI)**	Examiner	X						
**Screening for >25% bleeding on marginal probing**	Examiner	X						
**Bleeding on Marginal Probing[2] score (BOMP)**	Examiner		X	X	X	X	X	X
**Silness & Löe Plaque Index[3] (MPI)**	Examiner		X	X	X	X	X	X
**Gingival Abrasion Score[4](GAS)**	Examiner			X	X	X	X	X
**Enrollment**	Researcher	X						
**Randomization**	Researcher		X					
**Study Instructions**	Researcher	X	X	X	X	X	X	
**Hand out interdental devices**	Researcher		X		X			
**Hand out toothbrush and paste**	Researcher		X		X			
**Study Product Instruction**	Researcher		X			X		
**Supervised Product Use**	Researcher		X			X		
**Professional Prophylaxis**	Examiner			X				

**Safety Assessments**	Examiner		X	X	X	X	X	X
**Calendar, Dispense**	Researcher		X	X	X			
**Calendar, Collect**	Researcher			X	X			X
**Collect Interdental Devices**	Researcher			X				X
**Collect toothbrush and paste**	Researcher				X			X

[1]**DPSI**: 0-4: 0 = No pockets >3mm in depth, no calculus, no overhanging restorations and no bleeding on probing to the bottom of the pocket;1 = No pockets >3mm in depth, no calculus, no overhangs of restorations, but presence of bleeding on probing to the bottom of the pocket; 2 = No pockets >3mm in depth, presence of bleeding on probing to the bottom of the pocket, and presence of calculus or overhanging restorations;3 - = Presence of pockets of 4-5mm, bleeding on probing, supra en subgingival calculus and/or overhanging restorations without gingival recession;3 + = The same criteria as for score 3- but gingival recession; 4 = Presence of pockets ≥6mm.

[2]**BOMP:** 0-2: 0 = non-bleeding; 1 = pin prick; 2 = excess

[3]**MPI:** 0-3: 0 = No plaque; 1 = A film of plaque adhering to the free gingival margin and adjacent area of the tooth. The plaque may be seen in situ only after application of disclosing solution or by using the probe on the tooth surface; 2 = Moderate accumulation of soft deposits on the tooth and gingival margin that can be seen with the naked eye; 3 = Abundance of soft matter on the tooth and gingival margin.

[4]**GAS:** 3 categories: small, medium, large; Regions: molar, premolar, incisors + canines

Appendix Table 2. In- and exclusion criteria

**Inclusion criteria**
• Male and female
• Right handed brusher and writer
• Age 18-35 years
• Classified as systemically healthy, assessed by medical questionnaire
• Minimum of 20 natural teeth: at least 5 evaluable in each quadrant of the lower jaw
• Dutch Periodontal Screening Index (DPSI) 0 to 3- (appendix 14.3)
• ≥25% Bleeding On Marginal Probing (BOMP) in the lower jaw at the moment of clinical screening
• Waxed dental floss should fit interdentally in at least three interdental spaces per quadrant in the lower jaw, excluding the interdental central incisors space. Of these three spaces, at least two spaces should involve molar areas.
• Willing and able to give written informed consent
• Agree to follow the study instructions for the duration of the study
• Agree to refrain from brushing the lower jaw for 21 days in the experimental gingivitis phase
**Exclusion Criteria**
• Overt dental caries
• Usage of (>1 time a week) any interdental device other than the one assigned as part of regular daily oral care
• Smokers (Lie et al. 1998, definition non-smoker: no cigarette smoked for at least one year)
• Removable (partial) dentures
• Crowns, bridges and implant supported restorations in the lower jaw
• Overhanging restorations in the lower jaw as assessed with a periodontal probe
• Removable night guard
• Oral and/or peri-oral piercings
• Apparent oral lesions
• Presence of orthodontic banding (except for lingual retention wire)
• Oral surgery within the last 2 months
• Dental student or dental professional
• Participation in a clinical study within the previous 30 days
• Self-reported pregnancy or breastfeeding
• Use of antibiotics during the last 3 months
• Need of antibiotic prophylaxis prior to dental treatment
• Use of anti-inflammatory drugs on a regular basis
• Show evidence of any (systemic) disease or condition that could be expected to interfere with examination or outcomes of the study

Appendix Figure 1. Flowchart Patient information

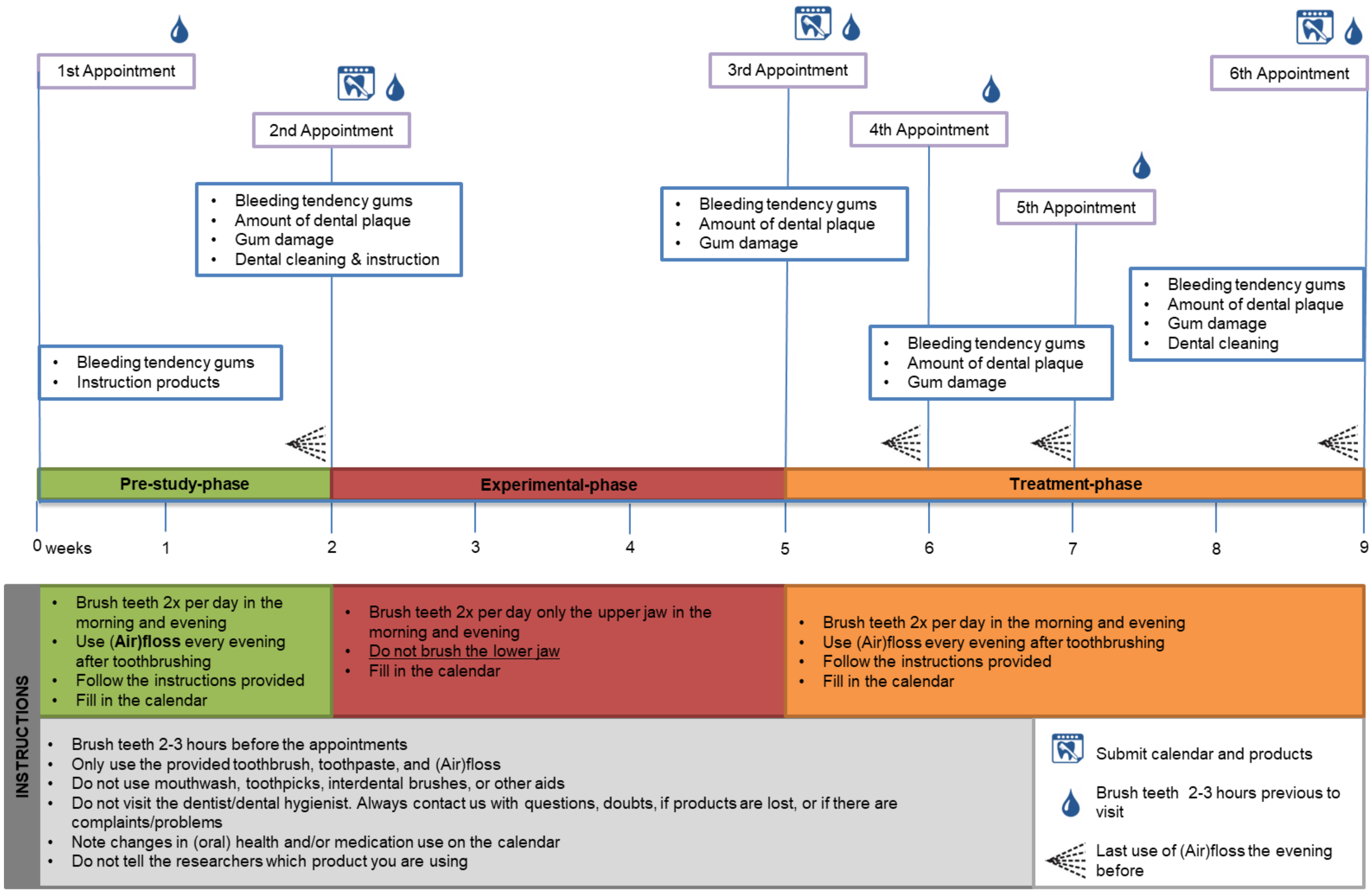

Appendix Figure 2. Boxplot presenting Bleeding On Marginal Probing (BOMP) scores from familiarization to treatment phase

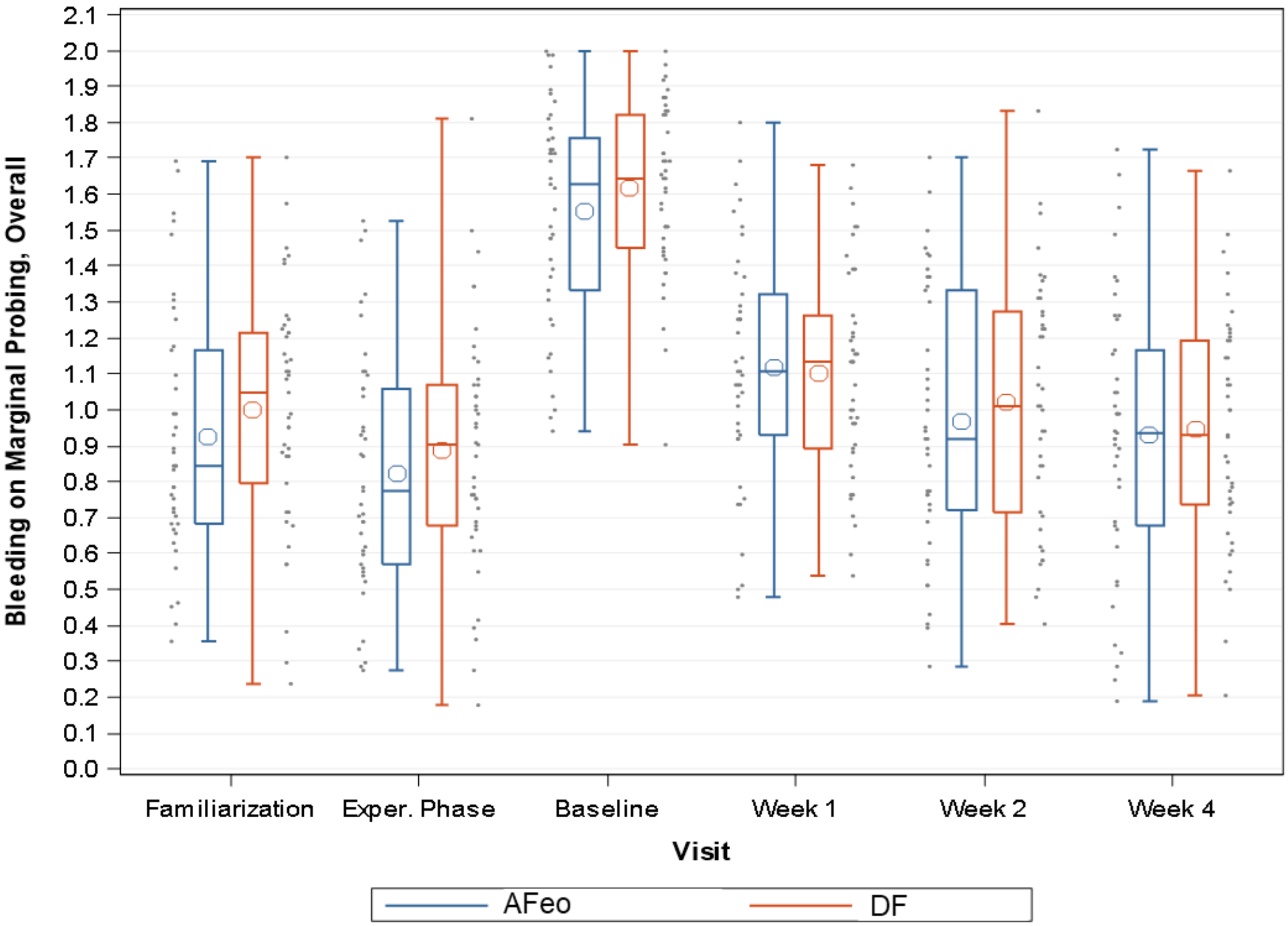

AFeo: AirFloss with essential oils

DF: Waxed dental Floss

Appendix Figure 3. Boxplot of the Modified Silness & Löe Plaque Index (MPI)

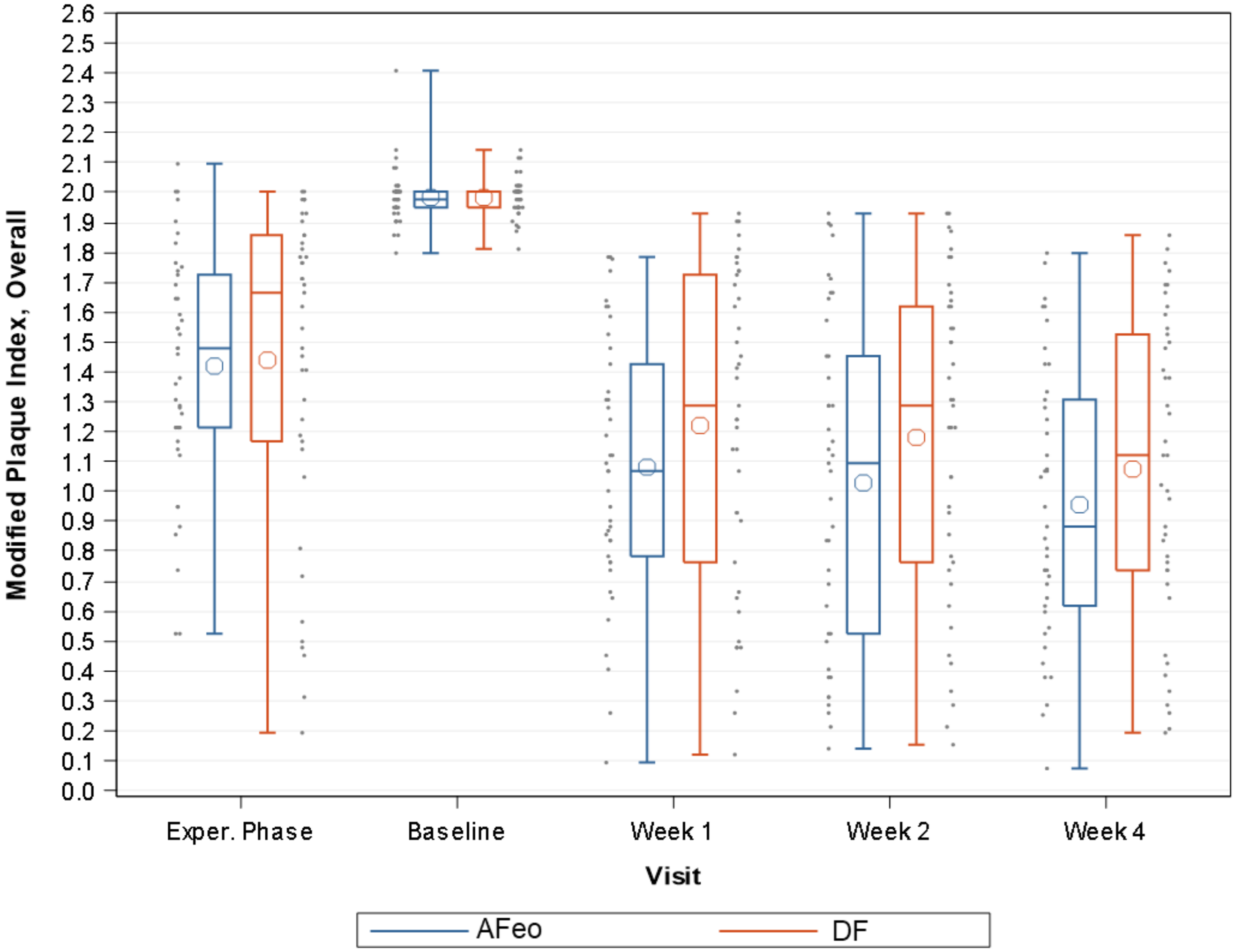

AFeo: AirFloss with essential oils

DF: Waxed dental Floss

Appendix Figure 4. Boxplot of the Gingival abrasion score (GAS).

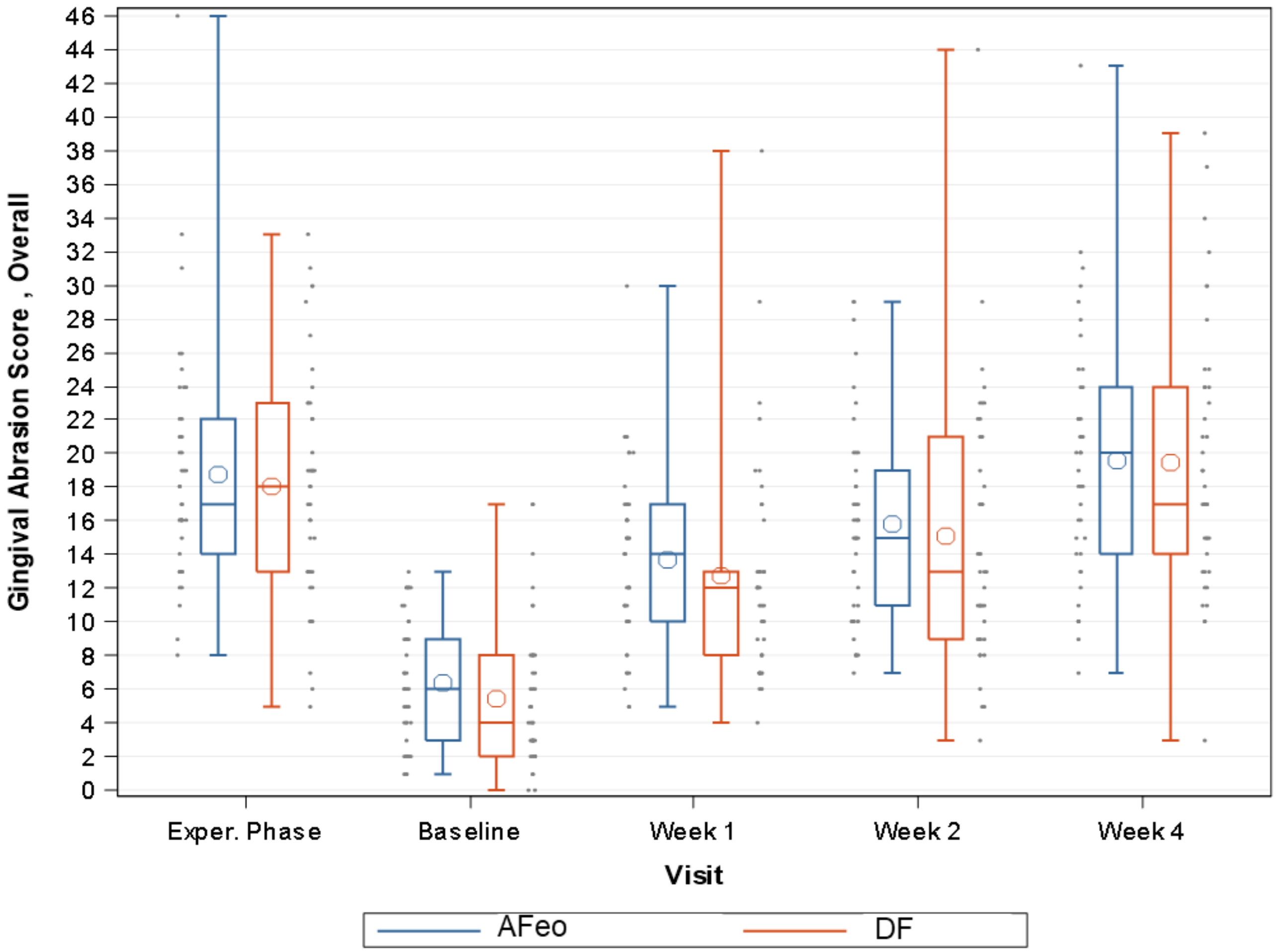

AFeo: AirFloss with essential oils

DF: Waxed dental Floss

Appendix A. Approval by the medical ethical committee at Amsterdam Medical Centre

Academisch Medisch Centrum
Universiteit van Amsterdam

Aan de heer prof.dr. G.A. van der Weijden
ACTA
ACTA Parodontologie
Gustav Mahlerlaan 3004
1081 LA Amsterdam

Amsterdam, 22 januari 2015
ons kenmerk: 2014_374#B201545
betreft: **Positief besluit**
NL51667.018.14
The ef[illegible]ro plus Listerine compared to dental floss on gingival bleeding, dental plaque, and gingival abrasion in a healing of experimental gingivitis model, a parallel design

Medisch Ethische Toetsingscommissie
E2-170
telefoon: 020 56 67389

Geachte heer Van der Weijden,

De METC AMC heeft zich, op grond van artikel 2, lid 2, sub a van de Wet medisch-wetenschappelijk onderzoek met mensen (WMO) beraden over bovengenoemd onderzoeksdossier.
Wij delen u gaarne mee dat onze commissie
- tot oordelen bevoegd krachtens artikel 2, tweede lid, onder a, van de Wet medisch-wetenschappelijk onderzoek met mensen (WMO);
- werkzaam volgens de ICH-GCP richtlijnen;
- op grond van de haar voorgelegde stukken als hierna vermeld;
- gelet op artikel 3 van de WMO;
- gelet op artikel 5 en 6;
- vastgesteld hebbende dat voorzien is in de dekking van een aansprakelijkheidsverzekering als bedoeld in artikel 7, lid 9 van de WMO,

heeft besloten tot een positief oordeel over deze studie en de uitvoering daarvan in de volgende centra: Academisch Centrum Tandheelkunde Amsterdam (ACTA).

Voorts hebben wij vastgesteld dat voor het onderzoek een verzekering is afgesloten conform de WMO door ACTA Dental Research B.V. ten behoeve van proefpersonen van het Academisch Centrum Tandheelkunde Amsterdam (ACTA).

In de beoordeling betrokken documenten:
A1 aanbiedingsbrief d.d. 4 december 2014
A1 aanbiedingsemail d.d. 5 december 2014
A1 aanbiedingsbrief d.d. 8 januari 2015
B1 ABR-formulier NL51667.018.14 versie 2 d.d. 8 januari 2015
C1 protocol versie 2 d.d. 8 januari 2015
D2 SPC listerine d.d. 10 september 2008
D2 SPC listerine d.d. 15 mei 2009
D2 SPC Philips AirFloss
E1 E2 proefpersoneninformatie en toestemmingsverklaring versie 2 d.d. 8 januari 2015 TC
E3 wervingsbrief bijlage tijdschema, ongedateerd
E3 wervingsbrief versie 2 d.d. 8 januari 2015
F1 gezondheidsvragenlijst versie 1 d.d. 4 december 2014
F2 patiëntendagboek poets en (air)floss kalender afspraak 1 naar 2 versie 2 d.d.8 januari 2015
F2 patiëntendagboek poets en (air)floss kalender afspraak 3 naar 6 versie 2 d.d. 8 januari 2015
F2 patiëntendagboek poets kalender afspraak 2 naar 3 versie 2 d.d. 8 januari 2015

F3 patiëntenkaart screening versie 2, ongedateerd
F3 patiëntenkaart visit 1 CRF versie 2, ongedateerd
F3 patiëntenkaart visit 2 CRF versie 2, ongedateerd
F3 patiëntenkaart visit 3 CRF versie 2, ongedateerd
F3 patiëntenkaart visit 4 CRF versie 2, ongedateerd
F3 patiëntenkaart visit 5 CRF versie 2, ongedateerd
F3 patiëntenkaart visit 6 CRF versie 2, ongedateerd
F4 patiënteninstructie tandfloss versie 2 d.d. 8 Januari 2015
G1 bewijs dekking WMO verzekering CentraMed VUMC_ACTA d.d. januari 2014
G2 bewijs dekking aansprakelijkheid Meijers UVA d.d. 12 november 2014
H1 CV onafh. arts Danser, ACTA
I2 onderzoeksverklaring ACTA d.d. 4 december 2014
I3 CV hoofdonderzoeker Van der Weijden, ACTA
K3 onderzoekscontract ACTA Dental Research B.V. d.d. 4 december 2014
K6 brief aan tandarts, ongedateerd
K6 adverse events log, ongedateerd
K6 memorandum of understanding Philips ADR d.d. 9 december 2014

Het onderzoeksdossier, aan ons ter beoordeling voorgelegd op 8 december 2014, is besproken in de vergadering van onze commissie van 18 december 2014 en vervolgens aan de orde geweest in de vergadering van het dagelijks bestuur van onze commissie van 20 januari 2015. Het dagelijks bestuur heeft geconstateerd, hiertoe gemandateerd door de commissie, dat met het voorleggen van de aangepaste stukken d.d. 9 januari 2015 de vragen van de commissie zijn beantwoord.

U dient onze commissie op de hoogte te stellen van de daadwerkelijke start van het onderzoek, van de (al dan niet voortijdige) beëindiging daarvan, en van tijdens de studie optredende onverwachte complicaties. Voorts dienen eventuele protocolwijzigingen ter beoordeling aan onze commissie te worden voorgelegd. U dient tevens ons jaarlijks een voortgangsrapportage betreffende de studie te doen toekomen, voor het eerst binnen een jaar na dagtekening van dit besluit.

Wij wijzen u erop dat op grond van artikel 23 van de Wet medisch-wetenschappelijk onderzoek met mensen degene wiens belang rechtstreeks bij een besluit van de METC is betrokken, daartegen binnen zes weken na de dag waarop het besluit bekend is gemaakt, een administratief beroepschrift kan indienen bij de Centrale Commissie Mensgebonden Onderzoek. Een dergelijk administratief beroepschrift dient geadresseerd te worden aan: CCMO, Postbus 16302, 2500 BH Den Haag.

Tenslotte brengen wij onder uw aandacht dat dit besluit zijn geldigheid verliest als de studie niet binnen één jaar na dagtekening van deze brief is gestart.

Ten tijde van de beoordeling van dit project was de commissie als volgt samengesteld:

prof.dr. M.P.M. Burger	:	voorzitter, gynaecoloog
mw.drs. G.H.M. van Ammers	:	lid dat onderzoek beoordeelt vanuit de invalshoek van de proefpersoon
dr. A.J. Bredenoord	:	maag darm lever arts
dr. M.G.W. Dijkgraaf	:	plv. lid, methodoloog
mw. J.M.M. Dijkstra	:	lid dat onderzoek beoordeelt vanuit de invalshoek van de proefpersoon
dr. M. Figee	:	psychiater
prof.dr. R.C.M. Hennekam	;	hoogleraar kindergeneeskunde en klinische genetica
prof.dr. J.J. Homan van der Heide	:	internist
dr. J.M.N.E. Jans	:	plv. lid, ethicus
dr. J.Ph. de Jong	:	plv. ethicus
dr. R.E. Jonkers	:	longarts/klinisch farmacoloog
prof.dr. A.H.L.C. van Kaam	:	kinderarts
mw.dr. E.M. Kemper	:	ziekenhuisapotheker, klinisch farmacoloog
dr. M.J.W. Koelemay	:	vaatchirurg
mw.dr. S.J. de Kort	:	plv. lid, medisch ethicus
prof.mr.dr. J. Legemaate	:	plv. lid, hoogleraar gezondheidsrecht
prof.dr. R.A.A. Mathôt	:	ziekenhuisapotheker, klinisch farmacoloog
dr. G.A. van Montfrans	:	internist
mw.dr. W.M.C. Mulder	:	plv. lid, klinisch farmacoloog
dr. P.J Nederkoorn	:	neuroloog
dr. M. Nieuwdorp	:	internist
mw.mr.dr. M.C. Ploem	:	gezondheidsjurist
dr. G. ter Riet	:	plv. lid methodoloog
dr. N.W.L. Schep	:	traumachirurg
prof.dr. A.J.P.M. Smout	:	hoogleraar gastroenterologie

Academisch Medisch Centrum
Universiteit van Amsterdam

mw.mr. L.M. Spittuler	:	lid dat onderzoek beoordeelt vanuit de invalshoek van de proefpersoon
dr. H.L. Tan	:	cardioloog
prof.dr. J.G.P. Tijssen	:	hoogleraar klinische epidemiologie van hart- en vaatziekten
mw.prof.dr. S. van de Vathorst	:	plv. lid, hoogleraar kwaliteit van de laatste levensfase en van sterven
prof.dr. M. Vermeulen	:	hoogleraar neurologie
prof.dr. D.L. Willems	:	hoogleraar medische ethiek
prof.dr. A.H. Zwinderman	:	plv. lid hoogleraar biostatistiek.

Voor de exacte samenstelling van de commissie tijdens de vergadering waarin het besluit is genomen, kunt u contact opnemen met het secretariaat van de commissie.

Met vriendelijke groet,
namens de Medisch Ethische Toetsingscommissie,

Mw. T. Groenveld,
ambtelijk secretaris

c.c. CCMO (pdf via TOL)
c.c. pdf per e-mail D.E.Slot

Appendix B. Dutch Periodontal Screening Index (DPSI)

Procedure

Dutch Periodontal Screening Index (DPSI) is a measure that functions as an initial screening evaluation to help estimate the level of periodontal disease involvement and gives direction to additional periodontal examination and subsequently the treatment needs of patients. It is a valuable tool for screening of the periodontal status (van der Velden 2009) and is assessed per sextant, and the Index is based on the site with the highest score.

Criteria

0 = No pockets >3mm in depth, no calculus, no overhanging restorations and no bleeding on probing to the bottom of the pocket

1 = No pockets >3mm in depth, no calculus, no overhangs of restorations, but presence of bleeding on probing to the bottom of the pocket

2 = No pockets >3mm in depth, presence of bleeding on probing to the bottom of the pocket, and presence of calculus or overhanging restorations

3 - = Presence of pockets of 4-5mm, bleeding on probing, supra en subgingival calculus and/or overhanging restorations without gingival recession

3 += The same criteria as for score 3- but gingival recession

4 = Presence of pockets ≥6mm.

References:

- Gómez SM, Danser MM, Sipos PM, Rowshani B, van der Weijden GA. Tongue coating and salivary bacterial counts in healthy/gingivitis subjects and periodontitis patients *J Clin Periodontol 2001*; **28**: 970-978.
- Van der Velden, U. The Dutch periodontal screening index validation and its application in The Netherlands. *J Clin Periodontol 2009*; **36**: 1018-1024U.

Appendix C. Gingival inflammation – Bleeding On Marginal Probing (BOMP)

For determining the level of gingival bleeding the gingivae are lightly dried with compressed air and lightly probed with a periodontal probe. The probe is inserted into the gingival crevice to a depth of approximately 2 mm or until slight resistance is felt. At this point the probe is run gently along the marginal gingiva holding the probe at an angle of approximately *60˚* and in contact with the sulcular epithelium. Minimal axial force is used to avoid undue penetration in the tissue. The probe is moved around the crevice gently stretching the epithelium. The probe is moved with a continuous motion along the entrance of the sulcus into the next interproximal area at both the vestibular and lingual surfaces. A WHO approved ball-ended probe (Ash Probe EN15, Dentsply International, York, PA, USA) will be used.

Method

A bleeding score is given to six gingival areas of the tooth. These are the disto-vestibular, vestibular, mesio-vestibular, disto-lingual, lingual and the mesio-lingual regions. First the vestibular surface is probed and scored. Thereafter the lingual surface is probed and scored. For each subject the number of bleeding points elicited are totalled and divided by the units probed. The index has a three point scale (0-2) to describe the bleeding tendency on the buccal or lingual aspects of each tooth.

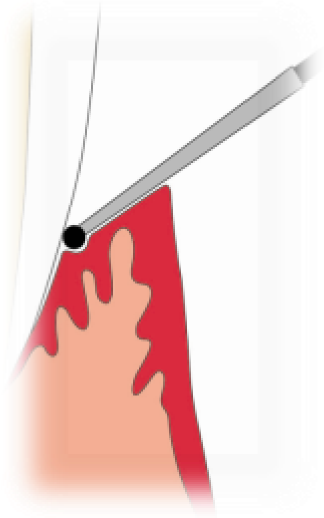

Bleeding on Marginal Probing (BOMP) score

The number of gingival units which bleed upon probing are recorded (scores 0, 1 and 2) Bleeding is scored within 30 seconds after probing.

0 = non-bleeding
1 = pin prick
2 = excess

References:

- Lie, M.A., Timmerman, M.F., Van der Velden, U. & Van der Weijden, G.A. (1998) Evaluation of 2 methods to assess gingival bleeding in smokers and non-smokers in natural and experimental gingivitis. Journal of Clinical Periodontology 25, 695-700.
- Saxton CA, van der Ouderaa FJ. The effect of a dentifrice containing zinc citrate and Triclosan on developing gingivitis. Journal Periodontal Research 1989;24 :75-80.

- Van der Weijden, G.A., Timmerman, M.F., Saxton, C.A., Russell, J.I., Huntington, E. & Van der Velden, U. (1994) Intra-/ Inter-examiner Reproducibility Study of Gingival Bleeding. Journal Periodontal Research 29, 236-241.
- Van der Weijden, G.A., Timmerman, M.F., Reijerse, E., Nijboer, A. & Van der Velden, U. (1994) Comparison of Different Approaches to Assess Bleeding on Probing as Indicators of Gingivitis. Journal Clinical Periodontology 21, 589-594.

Appendix D. Dental plaque - Modified Silness & Löe Plaque Index

The Silness & Löe Plaque Index, developed in 1964, is a widely used clinical tool for assessing dental plaque accumulation. This index evaluates the thickness of plaque at the gingival margin of teeth on a scale from 0 to 3, with higher scores indicating greater plaque presence. It is commonly used in both research and clinical settings to monitor oral hygiene and its impact on periodontal health.

Method

The Silness & Löe Plaque Index (Silness & Löe 1964) was modified as described by Van der Weijden et al. (1993a)), to score six surfaces on each tooth, rather than four surfaces. The surfaces are: distal-buccal, buccal, mesial-buccal, distal-lingual, lingual and mesial-lingual. Each of the six surfaces of the teeth is given a score of 0, 1, 2 or 3, according to the criteria.

Plaque index

0 = No plaque

1 = A film of plaque adhering to the free gingival margin and adjacent area of the tooth. The plaque may be seen in situ only after application of disclosing solution or by using the probe on the tooth surface

2 = Moderate accumulation of soft deposits on the tooth and gingival margin that can be seen with the naked eye

3 = Abundance of soft matter on the tooth and gingival margin

References:

- Danser MM, Timmerman MF, IJzerman Y, Piscaer M, van der Velden U, van der Weijden GA: Plaque removal with a novel manual toothbrush (X-Active) and the Braun Oral-B 3D Plaque Remover. J Clin Periodontol 2003; 30: 138–144.
- Van der Weijden GA, Danser MM, Nijboer A, Timmerman MF, Van der Velden U. The plaque-removing efficacy of an oscillating/rotating toothbrush. A short term study. J Clin Periodontol 1993; 273-278.
- Silness J and Löe H. Periodontal disease in pregnancy (II). Correlation between oral hygiene and periodontal condition. *Acta Odontol Scand* 1964;22:121-135.

Appendix E. Gingival abrasion score

The Gingival Abrasion Score is a clinical tool used to assess the presence and severity of abrasions or trauma to the gingival tissues. The score quantifies visible injury to the gingiva, often caused by improper brushing techniques or abrasive oral hygiene products. It is used in research to evaluate the impact of oral hygiene behaviors on gingival health.

Prior to staining the gingiva will be dried with the air blast and subsequently stained with Mira-2-Ton. Dye (non diluted) will be applied by the examiner with a fully saturated cotton swab starting from the lingual surfaces of the lower jaw (up to 1.5 cm from the gum line). For all assessment the same staining sequence will be followed. After staining, subjects will be instructed to rinse out their mouth with water (one sip only) and spit out very carefully.

Method

Prior to the assessment, the gingiva will be dried with the air blast. During the measurement the size of each lesion (colored dark blue) will be measured using periodontal probe (assessing the largest width of lesion). Loosely attached discolorations will be excluded from evaluation. If examiner has difficulty with the assessment at the gingival margin (plaque or abrasion), she/he will carefully try to remove the staining (non removable staining will be assesses as abrasion).

Abrasion score

Abrasions are split into 3 categories

- small, if Ø ≤ 2.5 mm,
- medium, if Ø > 2.5 mm, but ≤ 5 mm
- large, if Ø > 5 mm

Abrasions are recorded in the following regions (for each quadrant)

- M=molar
- P=premolar
- I=incisors + canine

For each region the approximal areas (interdental papillary-free gingiva) are assessed. The lesion in the approximal area in between two teeth (molar, premolar and incisor) is assigned to the closest tooth area. In each quadrant both surfaces (Lingual and Buccal) are assessed.

Each subject will be assigned a total number of small, medium and large lesions (separately) across all scorable sites. In addition, an overall total will be computed by adding together the number of small, medium and large lesions.

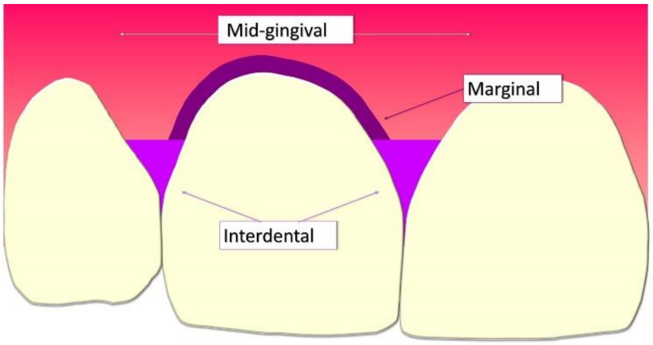

References:

- Breitenmoser, J., Mörmann, W. & Mühlemann, H.R. (1979) Damaging effects of toothbrush bristle end form on gingiva. *Journal Periodontology*, **50**, 212-216.
- Danser, M.M., Timmerman, M.F., IJzerman, Y., Van der Velden, U., Warren, P.R. & Van der Weijden, F.A. (1998a) A comparison of electric toothbrushes in their potential to cause gingival abrasion of oral soft tissues. *American Journal Dentistry* **11** (Special Issue), S35-39.
- Danser, M.M., Timmerman, M.F., IJzerman, Y., Bulthuis, H., Van der Velden, U. & Van der Weijden, G.A. (1998b) Evaluation of the incidence of gingival abrasion as a result of toothbrushing. *Journal of Clinical Periodontology* **25**, 701-706.
- Van der Weijden, G.A., Timmerman. M.F., Versteeg, P., Piscaer, M., Van der Velden, U. (2004) High & Low brushing force in relation to efficacy and gingival abrasion. *Journal of Clinical Periodontology*; **8**: 620-624

Appendix F. Patient Dental Floss Instruction.

1. Begin with approximately 40 cm of dental floss, loosely winding the ends around your middle fingers, leaving a 10 cm gap between them. Hold the floss taut with about 3 cm between your thumbs or create a loop.
2. Employ a gentle sawing motion as you guide the taut floss between front and back teeth, taking care in tight spaces to avoid snapping that could harm the gums.
3. Form a "U" shape around one tooth, pressing firmly against the side, and carefully slide the floss just under the gum using an up and down motion.
4. Draw the floss up to the proximal contact, then repeat the process on the adjacent tooth bordering the gum-filled space.
5. Safely remove the floss with a sawing motion and repeat the entire process for all other spaces in the mouth.
6. Ensure hygiene by using a fresh section of floss for each space, unwinding from one middle finger while winding it around the other.

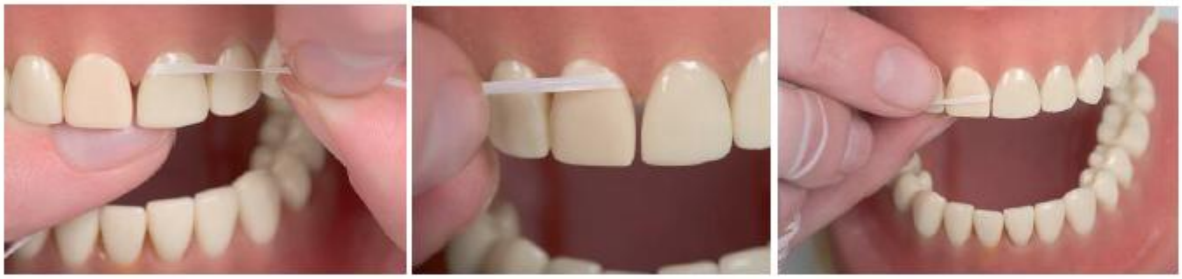

The original instructions are sourced from "Mechanical Supragingival Plaque Control" by F. van der Weijden and D. E. Slot, as found in Lindhe's Clinical Periodontology and Implant Dentistry, 7th Edition, Chapter 28, edited by T. Berglundh, with W. V. Giannobile, N. P. Lang, and M. Sanz.

Appendix G. Patient AirFloss Instruction.

Materials:

The AirFloss consists of three parts: a handheld device, the green tip, and a charger.
The handheld device is already charged, but this should be maintained by placing the device on the charger. A flashing green light indicates that the battery is charged, while a rapidly flashing yellow light signals that the device needs to be charged. A fully charged device can be used for approximately two weeks.

Preparations:

1. Attach the tip to the handheld device. It should click into place securely
2. Open the reservoir in the handheld device, located in the upper section

3+4.Fill the reservoir with liquid (water or mouthwash, depending on the phase of the treatment. Filling can be done from a tap, bottle, spoon, or syringe. The reservoir holds about two teaspoons of liquid.

5. Turn on the device using the button at the bottom of the handheld device
6. Aim the tip at the sink and press the green button six times

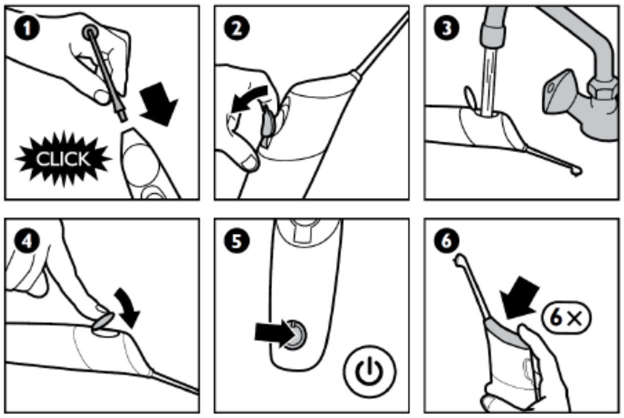

Use in the Mouth:

The AirFloss should be used once a day after brushing in the evening. Lean forward over the sink and place the AirFloss in your mouth. The AirFloss is used on the outer side of the teeth and molars.

1. Aim the tip at the teeth and place it between two molars and/or teeth, close to the gumline

2. Activate the AirFloss by briefly pressing the green button once. A short burst of air and liquid will be released from the tip.

3. Slightly close your lips to prevent splashing, but ensure the AirFloss remains properly positioned between two teeth or molars. Some spraying may occur in the mouth, and you can either let the liquid flow out or spit it into the sink. Move the tip along the gumline to the next space between two teeth or molars and press the green button again.

Press the green button once for each space between two teeth or molars. Ensure you hold the device upright and not upside down.

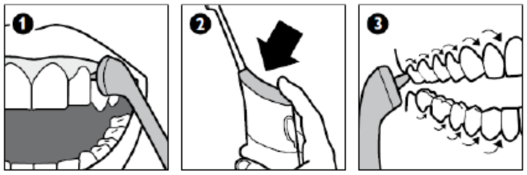

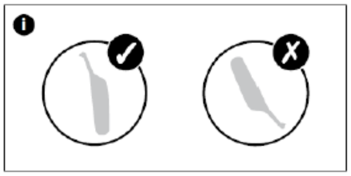

Sequence:

1. Start on the outer side of the upper right jaw, at the last space between two molars and/or teeth. Follow the gumline toward the front teeth.

2. Continue on the upper left side, starting at the last space between two molars and/or teeth, and move toward the front teeth.

3. Move to the outer side of the lower right jaw, starting from the back molars and working toward the front teeth.

4. Finally, clean the lower left side, starting at the back molars and moving toward the front teeth.

Cleaning:

After each use, the AirFloss must be cleaned.

Turn off the device.

1. Remove the tip from the handheld device. Rinse the tip under running water and dry it.

2. Open the reservoir in the handheld device

3. and rinse it with water.

4. Do not use dish soap or any other cleaning agents. Dry the handheld device.

5. When necessary, place the device on the charger.

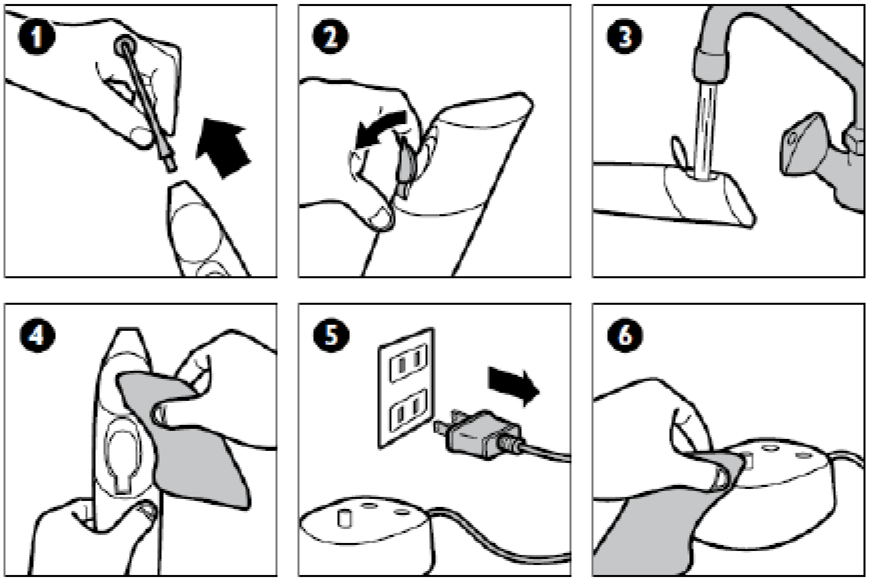

Supplement: Supplementary file 1 — Appendix Table 1: Table of events. Appendix Table 2: In‐ and exclusion criteria. Appendix Figure 1: Flowchart patient information. Appendix Figure 2: Boxplot presenting Bleeding On Marginal Probing (BOMP) scores from familiarisation to treatment phase. Appendix Figure 3: Boxplot of the Modified Silness and Löe Plaque Index (MPI). Appendix Figure 4: Boxplot of the Gingival abrasion score (GAS). Appendix A: Approval by the medical ethical committee at Amsterdam Medical Centre. Appendix B: Dutch Periodontal Screening Index (DPSI). Appendix C: Gingival inflammation—Bleeding On Marginal Probing (BOMP). Appendix D: Dental plaque—Modified Silness and Löe Plaque Index. Appendix E: Gingival abrasion score. Appendix F: Patient dental floss instruction. Appendix G: Patient AirFloss instruction. [file IDH-24-3-s001.pdf]
